# Genetic and Molecular Characterization of Treacher Collins Syndrome in Three Mexican Families

**DOI:** 10.3390/ijms27041891

**Published:** 2026-02-16

**Authors:** Saul Camarillo-Benitez, Claudia Fabiola Mendez-Catala, Maria del Carmen Chima-Galan, Claudia Rebeca Rivera-Yañez, Nancy Negrete-Torres, Teyda Anaid Arrieta, Julio Raul Alcantara-Torres, Adolfo René Méndez-Cruz, María Isabel Mendoza-Ramos, Norma Iliana Tapia-Soto, Efraín Garrido, Alexander Pedroza-Gonzalez, Gina Stella Garcia-Romo, Julia Reyes-Reali, Luis O. Soto-Rojas, Glustein Pozo-Molina, Dante Amato

**Affiliations:** 1 Laboratorio de Genética y Oncología Molecular, Carrera de Médico Cirujano, Facultad de Estudios Superiores Iztacala, Universidad Nacional Autónoma de México, Laboratorio 5, Edificio A4, Tlalnepantla 54090, Mexico; cabesamc@outlook.com (S.C.-B.); mendezcatalacf@gmail.com (C.F.M.-C.); drarrivera@unam.mx (C.R.R.-Y.); nadeneto19@live.com (N.N.-T.); arrietateyda@gmail.com (T.A.A.); jalcantara832@gmail.com (J.R.A.-T.); 2 División de Investigación y Posgrado, Facultad de Estudios Superiores Iztacala, Universidad Nacional Autónoma de México, Tlalnepantla 54090, Mexico; 3 Servicio de Genética Médica, Centro Médico Nacional “20 de Noviembre”, Instituto de Seguridad y Servicios Sociales de los Trabajadores del Estado, Ciudad de Mexico 03229, Mexico; carmenchimag@yahoo.com.mx; 4 Laboratorio de Inmunología, Unidad de Morfofisiología y Función, Facultad de Estudios Superiores Iztacala, Universidad Nacional Autónoma de México, Tlalnepantla 54090, Mexico; armendez@unam.mx (A.R.M.-C.); merisam06@iztacala.unam.mx (M.I.M.-R.); alexander_pg@yahoo.com.mx (A.P.-G.); garciaromogina@gmail.com (G.S.G.-R.); reali@unam.mx (J.R.-R.); 5 Carrera de Médico Cirujano, Facultad de Estudios Superiores Iztacala, Universidad Nacional Autónoma de México, Tlalnepantla 54090, Mexico; nitsrita@iztacala.unam.mx; 6 Departamento de Genética y Biología Molecular, Centro de Investigación y de Estudios Avanzados del Instituto Politécnico Nacional, Ciudad de México 07360, Mexico; egarrido@cinvestav.mx; 7 Laboratorio de Investigación en Neurociencias y Enfermedades Neurodegenerativas (LINEN), Carrera de Médico Cirujano, Facultad de Estudios Superiores Iztacala, Universidad Nacional Autónoma de México, Tlalnepantla 54090, Mexico; oskarsoto123@unam.mx

**Keywords:** *TCOF1*, *POLR1D*, *POLR1C*, *POLR1B*, TCS, Treacher Collins syndrome, whole exome sequencing, ribosomopathies

## Abstract

Treacher Collins syndrome (TCS) is a rare disorder within the group of mandibulofacial dysostoses, occurring in 1 in 50,000 live births. It is characterized by anomalies in the maxillary, mandibular, and stapes bones, among others. TCS is caused by pathogenic variants in the *TCOF1*, *POLR1D*, *POLR1C*, and *POLR1B* genes with autosomal dominant or recessive inheritance patterns. Genetic data from Latin American populations remain scarce. Eleven patients from three different families were recruited. Whole-exome sequencing (WES) was performed on the probands to identify genetic variants, followed by Sanger sequencing for variant validation and familial segregation analysis. Finally, three-dimensional protein structures of wild-type and mutant proteins were predicted. In Family 1, a heterozygous pathogenic splice-site variant in the *TCOF1* gene, c.4345 + 1 G > A, was identified and inherited from her mother. In Family 2, a heterozygous pathogenic variant in the *TCOF1* gene, c.226_227insC (p.R77fs), was identified and inherited from the paternal lineage. In Family 3, a heterozygous pathogenic *POLR1D* variant, c.290_291delAG (p.G99fs), was identified among multiple affected relatives; direct parent-of-origin could not be established due to unavailability of one parent, but segregation supports autosomal dominant transmission across three generations. All findings were validated by Sanger sequencing. Our findings highlight the utility of WES for the molecular diagnosis of TCS and underscore the importance of including underrepresented populations in genetic studies to improve diagnosis, genetic counseling, and perinatal planning in at-risk pregnancies.

## 1. Introduction

The exponential advancement of next-generation sequencing (NGS) technologies has revolutionized the landscape of genetic diagnosis and variant detection. Beyond variant detection, WES facilitates the discovery of previously unreported disease-causing variants and expansion of gene–phenotype correlations, progressively refining our understanding of genetic heterogeneity in rare craniofacial disorders [1,2,3].

Craniofacial anomalies account for approximately one-third of all congenital alterations, reflecting the complexity and developmental sensitivity of craniofacial morphogenesis. Clinically, facial dysostoses (FD) represent a group of etiologically heterogeneous craniofacial abnormalities subdivided into two major classifications: (1) mandibulofacial dysostoses (MFD), which present with craniofacial abnormalities exclusively, including hypoplasia of structures derived from the first and second pharyngeal arches such as the maxilla, mandible, zygomatic complex, and middle ear ossicles; and (2) acrofacial dysostoses (AFD), which are characterized by craniofacial features similar to MFD accompanied by distinctive limb anomalies [4,5,6]. Within the MFD group, Treacher Collins syndrome (TCS) stands out as the most representative and thoroughly characterized condition, a rare autosomal dominant genetic disorder characterized by bilateral craniofacial abnormalities. The incidence is reported as 1 in 50,000 live births, with no significant geographic variation, and notably, more than 50% of cases result from de novo mutations rather than hereditary transmission [7,8,9,10].

To date, four genes have been implicated in the genetic etiology of Treacher Collins syndrome, each contributing distinctly to disease pathogenesis based on inheritance patterns and relative mutational frequency. TCS1 (OMIM 154500) results from pathogenic variants in the *TCOF1* gene, which encodes treacle ribosome biogenesis factor 1, a nucleolar protein essential for coordinating RNA polymerase I-mediated rRNA transcription and ribosome biogenesis. *TCOF1* mutations follow an autosomal dominant (AD) pattern of inheritance. They are responsible for approximately 63–93% of molecularly diagnosed TCS cases, with the majority of pathogenic variants arising as de novo mutations in 60% of cases, reflecting a high rate of de novo occurrence. Mutations in the *POLR1D* gene cause TCS2 (OMIM 613717), encoding RNA polymerase I and III subunit D (POLR1D), and it accounts for approximately 6–8.4% of TCS cases, exhibiting either autosomal dominant or autosomal recessive (AR) inheritance patterns. TCS3 (OMIM 248390) involves pathogenic variants in the *POLR1C* gene, which encodes RNA polymerase I and III subunit C (POLR1C), with AR inheritance and represents approximately 1.05–1.2% of genetically confirmed cases. TCS4 (OMIM 618939), the most recently identified subtype, is caused by mutations in the *POLR1B* gene, encoding RNA polymerase I subunit B (POLR1B), exhibits autosomal dominant inheritance, and accounts for approximately 1.3–1.84% of cases. All four genes encode proteins directly or indirectly involved in rRNA transcription and ribosome biogenesis, suggesting that impaired ribosomal protein synthesis during neural crest cell differentiation and proliferation represents the fundamental pathogenic mechanism underlying craniofacial abnormalities in all TCS variants [8,11,12].

The cardinal craniofacial features of TCS can be stratified by prevalence into primary and secondary manifestations. Primary manifestations (occurring in >78% of affected individuals) include downward-slanting palpebral fissures (89–100%), malar hypoplasia and zygomatic complex hypoplasia (81–97%), bilateral conductive hearing loss (83–92%), and mandibular hypoplasia with micrognathia and retrognathia (78–91%), contributing to posterior airway narrowing and potential airway compromise, particularly in neonates. Secondary manifestations (occurring in 24–77% of cases) include atresia or stenosis of the external auditory canal (68–71%); microtia (10–77%); coloboma or notching of the lower eyelid (54–69%); and delayed speech and language development (57–63%). Notably, cognitive development is typically normal in TCS [8,12,13,14].

The pathogenesis of TCS is classified as a ribosomopathy, a rare genetic disorder characterized by disrupted ribosome biogenesis. In TCS, mutations in *TCOF1*, *POLR1D*, *POLR1C*, or *POLR1B* impair RNA polymerase I and III (Pol I/III) function, resulting in decreased transcription of pre-ribosomal RNA species (45S rRNA, 5S rRNA, and tRNAs), which compromises the assembly of 60S and 40S ribosomal subunits. This defect is particularly deleterious during craniofacial morphogenesis (embryonic weeks 3–8), when neural crest cells (NCCs) undergo rapid proliferation and directed migration from the first and second pharyngeal arches to generate craniofacial bone and cartilage. Since ribosome biogenesis is an exceptionally energy-demanding process accounting for approximately 80% of all nuclear transcription and up to 95% of total cellular RNA content, the reduction in ribosomal subunit assembly and protein synthesis capacity leads to nucleolar stress and p53-mediated apoptosis of NCCs. The selective vulnerability of craniofacial tissues reflects their extraordinary dependence on robust protein synthesis for sustaining the high proliferative and migratory demands of NCC differentiation [12,15,16,17,18,19,20]. Pathogenic variants that impair ribosome biogenesis can trigger nucleolar stress, allowing p53 to accumulate and repress RNA polymerase I-mediated rRNA transcription, which further worsens the biogenesis defect. Persistent p53 then induces p21/CDKN1A, arresting the cell cycle and activating pro-apoptotic programs that lead to apoptosis of affected neural crest cells [15,21,22,23,24,25].

Despite the identification of four disease-causing genes and characterization of their molecular functions in ribosome biogenesis, the mechanisms underlying phenotypic heterogeneity in TCS remain incompletely understood. While severe *TCOF1* mutations typically produce more pronounced craniofacial abnormalities, variable expressivity is frequently observed even among family members carrying identical mutations, suggesting the involvement of genetic modifiers. To address this knowledge gap and provide molecular insights into disease pathogenesis in understudied populations, we present a comprehensive clinical and molecular characterization of Treacher Collins syndrome in the Mexican population.

## 2. Results

### 2.1. Clinical Evaluation

**Figure 1:**
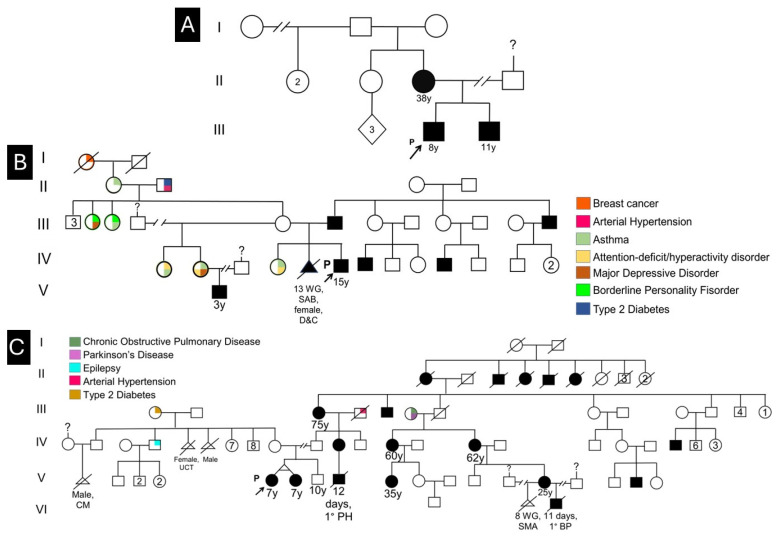
Pedigree of the three families studied. (**A**) Family 1 includes three patients with different clinical manifestations. Patient 1 (III:4). Patient 2 (III:5). Patient 3 (II:4). (**B**) Family 2 comprises three patients with varied clinical manifestations. Patient 4 (IV:6). Patient 5 (III:8). Patient 6 (V:1). (**C**) Family 3 consists of several patients with clinical manifestations of TCS. Patient 7 (V:7). Patient 8 (V:8). Patient 9 (V:11). Patient 10 (V:16). Patient 11 (IV:26). WG, weeks of gestation; SAB, miscarriage; D&C: dilatation and curettage; ↗ indicate probands (P), and the symbol “?” denotes an unknown or unverified familial branch. Each colored box indicates different diseases presented by different members of each family. All vector graphics were created using Inkscape v1.4.3 (Inkscape Project, https://inkscape.org; accessed on 18 December 2025). All graphics were processed using GIMP v3.0.8 (GIMP Development Team, https://www.gimp.org; accessed on 18 December 2025).

This study included eleven confirmed TCS patients from three unrelated Mexican families, encompassing clinical disease presentation ranging from severe to mild phenotypic manifestations. Of the eleven participants, nine had received a prior clinical diagnosis of TCS from specialized craniofacial or genetic teams but lacked molecular genetic confirmation; notably, two individuals were newly diagnosed through this study, revealing previously unrecognized disease based on phenotypic screening and subsequent molecular confirmation. The substantial phenotypic heterogeneity observed across the cohort, ranging from individuals presenting cardinal craniofacial features including malar hypoplasia, mandibular hypoplasia, and conductive hearing loss to those exhibiting more subtle or atypical manifestations, underscores the variable expressivity characteristic of TCS despite mutations in the same causative genes. Detailed clinical characterization of all 11 participants, including cardinal and secondary craniofacial features, audiological findings, and functional impairments, is provided in Table 1 and the pedigrees in Figure 1.

### 2.2. Genetic Analysis

Whole-exome sequencing achieved a mean on-target depth of ~100× (minimum ~80×). The mean read mapping rate was 94.1% ranging from 91.3% to 96.7%, and the optical duplicate rate was ~2.5%. Quality control by FastQC (https://www.bioinformatics.babraham.ac.uk/projects/fastqc/, accessed on 4 August 2025) confirmed base-level quality ≥ Q30. These metrics indicate sufficient sequencing depth and data quality for reliable variant detection. WES identified pathogenic variants in TCS-associated genes in 10 of 11 patients (90.9%). These include a canonical splice-site variant (*TCOF1* c.4345 + 1 G > A) and frameshift indels (*TCOF1* c.226_227insC; *POLR1D* c.290_291delAG). All sequence variants reported were absent from public databases (ClinVar (https://www.ncbi.nlm.nih.gov/clinvar/, accessed on 4 August 2025), gnomAD (https://gnomad.broadinstitute.org/, accessed on 4 August 2025), LOVD3 (https://www.lovd.nl/3.0/home), VarSome (https://varsome.com/), InterVar (https://wintervar.wglab.org/); accessed on 18 January 2026), and no prior primary-literature reports of the same HGVS description under the specified transcripts were identified.

#### 2.2.1. Family 1

**Figure 2:**
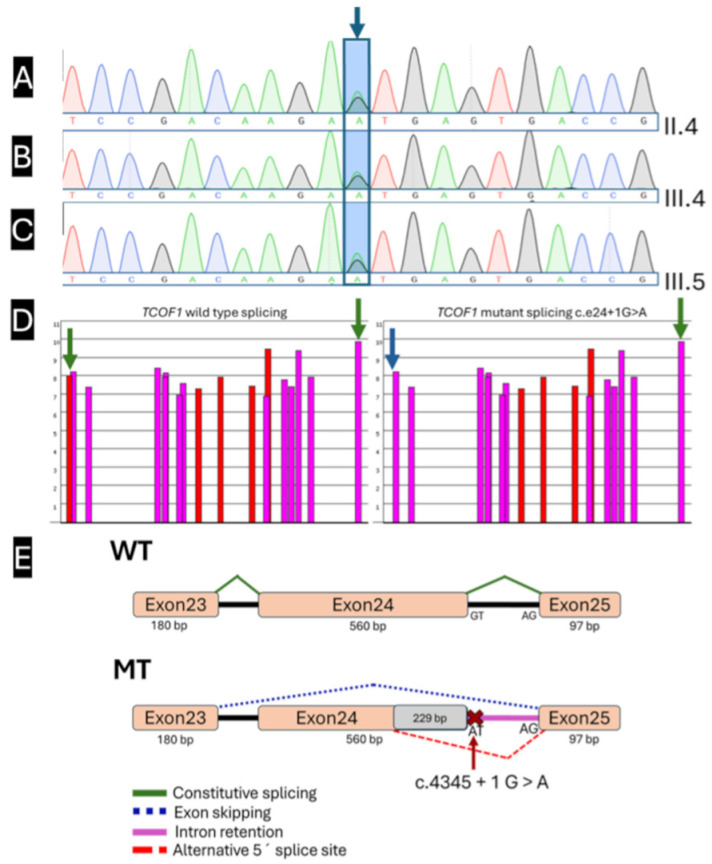
Molecular and in silico splicing analysis of the *TCOF1* c.4345 + 1 G > A variant. (**A**–**C**) Sanger sequencing electropherograms confirming the heterozygous canonical splice donor variant c.4345 + 1 G > A in intron 24 of *TCOF1* in three affected individuals: (**A**) Patient 3 (mother of the proband, individual II:4), (**B**) Patient 1 (proband, individual III:4), and (**C**) Patient 2 (brother of the proband, individual III:5). (**D**) In silico predictions of the wild-type (WT) at left and mutant (MT) on right splice site of exon 24 generated with ESEFinder 3.0; possible 5′ splice sites are shown in the red bar graph, possible 3′ splice sites are shown in the pink bar graph; green arrows represent the canonical splice sites, in c.4345 marked with a blue arrow represents a possible loss of donor splice site. (**E**) Comparative representation of predicted splicing patterns based on in silico analysis. In the WT transcript, exon 24 is correctly spliced between exons 23 and 25 (constitutive splicing; green line). In the MT context, loss of canonical donor site recognition is strongly predicted by SpliceAI (donor loss score = 0.99), with exon 24 skipping as the most likely aberrant outcome (blue dashed line). Additional predicted alternative splicing events include partial intron 24 retention (purple line) and activation of a cryptic donor splice site approximately 229 bp upstream within exon 24 (red dashed line). Electropherograms generated and exported with SnapGene v8.2.2 (free Viewer mode; Dotmatics, https://www.snapgene.com/snapgene-viewer, accessed on 4 August 2025). All vector graphics were created using Inkscape v1.4.3 (Inkscape Project, https://inkscape.org). All raster graphics were processed using GIMP v3.0.8 (GIMP Development Team, https://www.gimp.org).

A heterozygous canonical splice site variant, *TCOF1* (NM_001371623.1): c.4345 + 1 G > A (GRCh38 (https://www.ncbi.nlm.nih.gov/datasets/genome/GCF_000001405.26/, accessed on 4 August 2025): chr5: 150,396,843 G > A), affecting the canonical +1 donor of exon 24 (intron 24 boundary) (Figure 2A–E) was identified in Patient 1 (Figure 1A, pedigree III:4). This variant was present in Patient 1 and two affected relatives, consistent with an autosomal dominant inheritance pattern characteristic of TCS. Segregation analysis using Sanger sequencing validation confirmed the variant in all three patients. It was established that maternal inheritance from the heterozygous mother (Figure 1A, Patient 3, pedigree II:4) to her two affected children (Patient 1, pedigree III:4, and Patient 2, pedigree III:5), (Figure 1A and Figure 2A–C).

The predicted impact of this variant on pre-mRNA splicing was evaluated using three complementary splice prediction algorithms: SpliceAI (https://spliceailookup.broadinstitute.org/, accessed on 4 August 2025), Pangolin (https://spliceailookup.broadinstitute.org/, accessed on 4 August 2025), and ESEFinder 3.0, which employ independent methodologies. SpliceAI (https://spliceailookup.broadinstitute.org/, accessed on 4 August 2025) predicted donor site loss with a score of 0.99 (−1 bp), indicating ≥99% probability of abrogating normal donor site function at the canonical position (−1 bp from variant), and additionally predicted donor site gain with a score of 0.29 (−229 bp), suggesting a moderate probability of cryptic donor site activation approximately 229 bp upstream, potentially resulting in aberrant splicing (Figure 2D,E). Pangolin analysis corroborated donor site loss with a score of 0.84 (−1 bp), providing an independent high-confidence prediction of splicing disruption. At the same time, ESEFinder 3.0 demonstrated diminished exonic splicing enhancer (ESE) motif recognition in the mutant sequence compared to wild-type (Figure 2D,E), further supporting impaired splice site recognition.

According to ACMG/AMP Standards and Guidelines [26,27], this variant is pathogenic based on the following: PVS1 (very strong) disrupts the canonical +1 donor site, abolishing normal splicing and consistent with a known loss-of-function mechanism in *TCOF1*; PM2 (moderate) absent from population databases (gnomAD v2.1.1/v3.1.2, 1000 Genomes; accessed 18 January 2026); PP3 (supporting) concordant in silico splice predictions indicating donor-site loss; and PP4 (supporting) highly specific Treacher Collins phenotype. The variant was not found in clinical repositories (ClinVar, VarSome, LOVD3, InterVar, gnomAD; accessed 18 January 2026) (Table S1, Supplementary Material).

#### 2.2.2. Family 2

**Figure 3:**
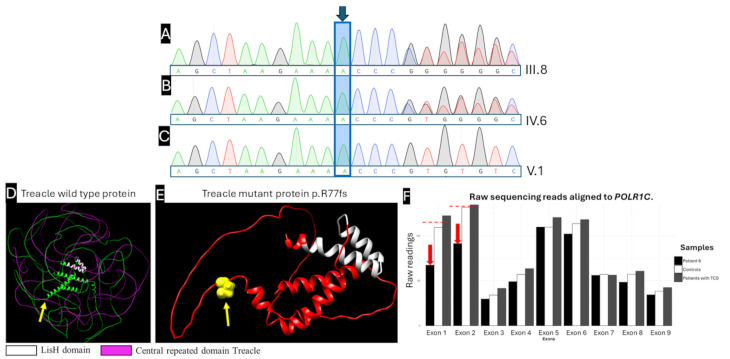
Analysis of the c.226_227insC (p.R77fs) genetic variant. Sanger sequencing results of exon 3: (**A**) Patient 4 (IV:6), (**B**) Patient 5 (III:8), and (**C**) Patient 6 (V:1). The heterozygous variant in *TCOF1* gene was confirmed in Patients 4 and 5. (**D**) AlphaFold2-predicted structure of wild-type treacle. The mutation site (Arg77) is annotated in yellow (arrow). The LisH domain is shown in white, and the central Treacle repeat domain is shown in pink. (**E**) AlphaFold2-predicted structure of the mutant treacle (p.Arg77Ilefs*97). The frameshift at Arg77 is annotated in yellow (arrow) and is predicted to cause premature truncation at ~aa 173 in the reference transcript, with loss of the central Treacle repeat domain (pink). The resulting truncated C-terminal segment is rendered in red; the LisH domain (white) is partially retained. (**F**) Comparative analysis of mean raw read counts across exons 1–9 in *POLR1C*, showing exon-level coverage in the affected individual (Patient 6, red arrows) compared with unaffected controls and patients with Treacher Collins syndrome carrying variants in other genes. Bars represent the average read counts per exon. The red dashed horizontal line indicates the expected mean read depth calculated from healthy controls and TCS controls included in the study, serving as a reference for comparative exon coverage assessment. (**D**,**E**): yellow = mutation-site residue; white = LisH domain; pink = central Treacle repeat domain (present in WT, absent in mutant); red = truncated C-terminal segment in the mutant model. Note: These are predicted structural models (used for visualization/interpretation alongside genetic evidence), not experimental structures. Electropherograms generated and exported with SnapGene v8.2.2 (free Viewer mode; Dotmatics, https://www.snapgene.com/snapgene-viewer, accessed on 4 August 2025). Protein three-dimensional structures were predicted with AlphaFold2 (ColabFold v1.5.5, https://colab.research.google.com/github/sokrypton/ColabFold/blob/main/AlphaFold2.ipynb, accessed on 4 August 2025), an open-source tool for high-accuracy protein structure prediction [28]. Predicted structures were obtained from the AlphaFold Protein Structure Database [29]. Three-dimensional visualization and rendering were performed using UCSF ChimeraX v1.8 (https://www.cgl.ucsf.edu/chimerax/, accessed on 4 August 2025). R Core Team. (2024). R: A language and environment for statistical computing (Version 4.3.3). R Foundation for Statistical Computing. All vector graphics were created using Inkscape v1.4.3 (Inkscape Project, https://inkscape.org). All raster graphics were processed using GIMP v3.0.8 (GIMP Development Team, https://www.gimp.org).

A heterozygous insertion variant, c.226_227insC (p.Arg77Ilefs*97), located in exon 3 of the *TCOF1* gene (NM_001371623.1), was identified in Patient 4 (pedigree IV:6). This variant causes a +1 nucleotide frameshift, resulting in translation of an altered reading frame through the premature introduction of a stop codon at amino acid position 173, generating a truncated protein of 173 amino acids compared to the wild-type full-length treacle (1489 aa, NP_001358552.1). The clinical presentation of Patients 4–6 was consistent with TCS phenotype. Sanger sequencing validation confirmed the insertion in Patients 4 and 5, establishing paternal inheritance from the heterozygous father (Patient 5, pedigree III:8) to his affected son (Figure 1B, Patient 4, pedigree IV:6) (Figure 3A–C).

The frameshift-induced structural consequences were assessed through AlphaFold2-based three-dimensional protein modeling, comparing the truncated mutant protein (NP_001358552.1:p.Arg77Ilefs*97, 173 amino acids, Figure 3E) with the wild-type full-length treacle (Figure 3D). Predicted models support disruption of N-terminal regions, including nucleolar localization signals and interfaces implicated in Pol I pre-initiation complex assembly; these in silico visualizations are supportive and interpreted alongside genetic evidence (PVS1).

According to ACMG/AMP guidelines, this variant is pathogenic based on the following: PVS1 (very strong) frameshift at the canonical coding sequence generates a premature termination codon at aa ~173, upstream of the final exon, consistent with nonsense-mediated decay and established loss-of-function mechanism in *TCOF1*; PM2 (moderate) absent from population databases (ExAC, gnomAD, 1000 Genomes; accessed 18 January 2026); and PP4 (supporting) clinical features highly specific for Treacher Collins syndrome. PM4 (protein length change) is not invoked to avoid double counting LOF evidence already captured by PVS1. The variant is not present in clinical repositories (ClinVar, VarSome, LOVD3, InterVar, gnomAD; accessed 18 January 2026) (Table S1, Supplementary Material).

Patient 6 remains molecularly unsolved; no pathogenic SNVs/indels were identified in *POLR1C*, *TCOF1*, or *POLR1D*. Exome read-depth suggests a heterozygous candidate CNV spanning *POLR1C* exons 1–2 (normalized coverage ~0.46 vs. diploid baseline). Read depth coverage was evaluated using the Integrative Genomics Viewer (IGV), which enables visual assessment and quantification of read counts across genomic regions, with normalized coverage values computed from alignment data. Quantitative read count analysis was performed by directly comparing Patient 6 with two reference cohorts: an external control dataset of 11 unrelated WES samples and 3 control samples from the current study, enabling detection of regional coverage deviations from expected diploid patterns. The consistent reduction in read depth across exons 1–2 of *POLR1C* in Patient 6 relative to all controls suggested a structural deletion affecting the 5′ coding region, a clinically relevant finding as mutations in *POLR1C* are classically associated with autosomal recessive forms of Treacher Collins syndrome (TCS3).

This heterozygous deletion in *POLR1C* exons 1–2 is considered a candidate CNV. Given the well-recognized limitations of WES for CNV detection, this finding requires orthogonal validation using an independent method like qPCR/MLPA. As *POLR1C*-related TCS is classically autosomal recessive, the identification of a single heterozygous CNV is insufficient to establish a molecular diagnosis; therefore, a second pathogenic allele would be required to establish molecular diagnosis in this case. In this patient, no probably pathogenic or pathogenic genetic variants were identified in the *TCOF1*, *POLR1D* and *POLR1C* genes (Table S2, Supplementary Material). No conclusions regarding penetrance or expressivity can be drawn in the absence of biallelic pathogenic variants or functional validation.

#### 2.2.3. Family 3

**Figure 4:**
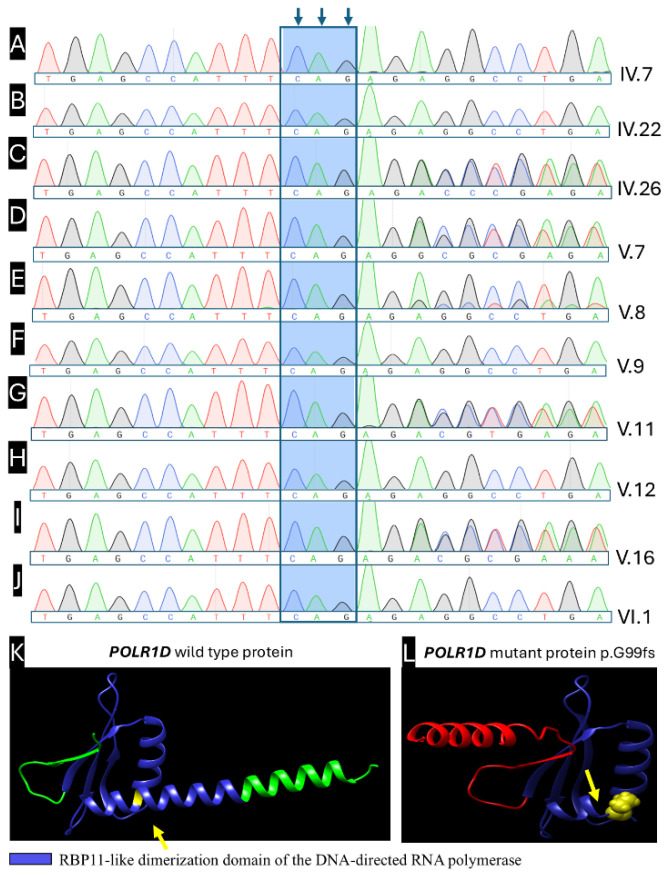
Analysis of the variant c.290_291delAG (p.G99fs) in *POLR1D* gene. The heterozygous variant in *POLR1D* gene in exon 2 was confirmed. (**A**) Unaffected relative (IV:7), (**B**) Unaffected relative (IV:22), (**C**) Patient 11 (IV:26), (**D**) Patient 7 (V:7), (**E**) Patient 8 (V:8), (**F**) Unaffected relative (V:9), (**G**) Patient 9 (V:11), (**H**) Unaffected relative (V:12), (**I**) Patient 10 (V:16), and (**J**) Unaffected relative (VI:1). (**K**) AlphaFold2-predicted structure of wild-type POLR1D. The RBP11-like dimerization domain (aa 39–112) is shown in blue. The Gly99 residue (mutation site) is annotated in yellow (arrow). The distal C-terminal segment is rendered in green. (**L**) AlphaFold2-predicted structure of the mutant POLR1D (p.Gly99Ilefs*2). The frameshift at Gly99 is annotated in yellow (arrow) and introduces a premature stop two codons downstream (predicted total length ~100 aa), thereby truncating the C-terminus and disrupting the RBP11-like dimerization domain (blue). The residual terminal segment is rendered in red for clarity. (**K**,**L**): blue = RBP11-like dimerization domain (aa 39–112); yellow = mutation-site residue (Gly99); green = wild-type distal segment; red = residual truncated segment in the mutant model. Note: Orientation differences between WT and mutant renderings reflect visualization of truncation and do not imply experimentally validated dynamic conformational changes. Electropherograms generated and exported with SnapGene v8.2.2 (free Viewer mode; Dotmatics, https://www.snapgene.com/snapgene-viewer, accessed on 4 August 2025). Protein three-dimensional structures were predicted with AlphaFold2 (ColabFold v1.5.5), an open-source tool for high-accuracy protein structure prediction [28]. Predicted structures were obtained from the AlphaFold Protein Structure Database [29]. Three-dimensional visualization and rendering were performed using UCSF ChimeraX v1.8 (https://www.cgl.ucsf.edu/chimerax/, accessed on 4 August 2025). All vector graphics were created using Inkscape v1.4.3 (Inkscape Project, https://inkscape.org). All raster graphics were processed using GIMP v3.0.8 (GIMP Development Team, https://www.gimp.org).

A heterozygous 2-nucleotide deletion, NM_015972.4:c.290_291delAG (NP_057056.1:p.Gly99Ilefs*2), located in exon 2 of the *POLR1D* gene (encoding RNA Polymerase I and III Subunit D), was identified in Patient 7 (Figure 1C, pedigree V:7). This variant causes a +1 frameshift mutation resulting in premature termination codon two codons downstream, yielding a severely truncated polypeptide of ~100 amino acids compared to the wild-type full-length *POLR1D* protein of 133 amino acids. The clinical presentations in Patient 7 and affected relatives were consistent with TCS phenotype. Segregation analysis demonstrated the presence of the *POLR1D* c.290_291delAG (p.Gly99Ilefs*2) variant among multiple affected individuals within the extended paternal family. The biological father of the proband was not available for molecular testing; therefore, direct parent-of-origin assignment could not be established. Sanger sequencing demonstrated vertical transmission consistent with autosomal dominant inheritance (Figure 4C–E,G,I).

The frameshift-induced structural impact was evaluated through AlphaFold2-based three-dimensional protein modeling, comparing the wild-type POLR1D structure (133 amino acids, Figure 4K) with the truncated mutant protein (p.Gly99Ilefs*2, ~100 amino acids, Figure 4L). The predicted structural models revealed severe disruption of the C-terminal segment, encompassing residues 101–133 that form the functionally critical POLR1C-binding interface (putative heterodimerization domain spanning residues 39–112). This structural deletion is predicted to abolish or severely impair heterodimerization between POLR1D and POLR1C, the essential heterodimeric unit required for assembly and function of RNA polymerase I and III complexes. These in silico visualizations are supportive and interpreted alongside genetic evidence (PVS1).

According to ACMG/AMP guidelines, this variant is pathogenic based on the following: PVS1 (very strong) frameshift introducing a premature stop, consistent with the established loss-of-function mechanism for POLR1D in Treacher Collins syndrome; PM2 (moderate) absent from population databases (ExAC, gnomAD, 1000 Genomes) and clinical repositories (ClinVar, LOVD3, InterVar, VarSome) at the time of curation; PP1 (supporting) co-segregation with disease in five affected relatives across three generations; and PP4 (supporting) phenotype highly specific for autosomal dominant TCS (malar/mandibular hypoplasia, external auditory canal atresia, conductive hearing loss). PM1/PM4/PP3 were not invoked to avoid double-counting LOF evidence or adding non-essential in silico support when PVS1 is definitive (Table S1, Supplementary Material).

The extended pedigree reveals multiple affected individuals with concordant craniofacial findings consistent with TCS, and the pattern of inheritance compatible with autosomal dominant transmission. The presence of the identical variant across five family members across three generations, combined with the observation of a single founder introduction of the variant, strongly suggests a founder event with subsequent vertical transmission, explaining the high penetrance and consistent phenotypic expression observed within this kindred. The absence of documented unaffected heterozygous carriers within the pedigree further supports complete or near-complete penetrance of this loss-of-function variant in the heterozygous state.

## 3. Discussion

This study provides the first comprehensive clinical–molecular characterization of Treacher Collins syndrome (TCS) in the Mexican population and identifies three novel pathogenic variants, underscoring the value of rigorous genetic diagnosis in rare craniofacial disorders expanding current knowledge of genetic heterogeneity and phenotypic diversity in understudied populations [8,30,31]. In our cohort, whole-exome sequencing achieved a diagnostic yield of 90.9% (10/11), a performance largely driven by phenotypic pre-selection of individuals meeting cardinal TCS criteria and by targeted analysis with orthogonal validation, consistent with prior evidence that focused ascertainment and curated pipelines increase yield relative to unselected cohorts [32,33,34,35]. Notwithstanding this yield, variant interpretation remains intrinsically challenging due to evolving evidence and occasional database inconsistencies; to mitigate this, we applied HGVS-conformant, transcript-aware curation with date-stamped queries across population and clinical repositories [2].

We identified three novel pathogenic variants across TCS-associated genes; by gene, *TCOF1* accounted for 45.5% of cases (Patients 1–5), *POLR1D* for 45.5% (Patients 7–11), and *POLR1C* represented a probable case in 9.1% (Patient 6, candidate heterozygous deletion). A transcript-aware ClinVar query (accessed 18 January 2026) returned 852 submissions for *TCOF1*, of which 201 (23.6%) are classified as pathogenic/likely pathogenic, with recurrent enrichment at exon 24; Family 1 splice-site variant maps to this hotspot. Family 2 harbors a pathogenic variant in exon 3 of *TCOF1*, to our knowledge the first exonic event reported at this locus, expanding the 5′ mutational spectrum. For *POLR1D*, ClinVar lists 121 entries with a recurrent exon 3 cluster; the Family 3 frameshift contributes to this cluster [36,37].

Patients carrying identical pathogenic variants frequently exhibit marked phenotypic divergence, a phenomenon consistently reported across TCS studies and that precludes the establishment of robust genotype–phenotype correlations. This observation underscores one of the most remarkable aspects of TCS biology: phenotypic severity and variable expressivity are not determined solely by the primary causal variant but are modulated by genetic modifiers, environmental influences, and stochastic developmental events. Mouse model studies provide mechanistic insights into this phenomenon: genetic background substantially modulates TCS disease severity, with studies demonstrating that strain-specific variation in endogenous treacle abundance and reactive oxygen species (ROS) homeostasis correlates inversely with phenotypic involvement, that is, strains with higher basal treacle levels and lower ROS exhibit milder phenotypes. Notably, Patients 7 and 8 are monozygotic twins harboring the same *POLR1D* pathogenic variant. The discordance in clinical severity between these genetically identical individuals underscores the role of modifying influences including epigenetic mechanisms, developmental stochasticity, environmental exposures, and genetic-background effects in shaping Treacher Collins expressivity. Evidence from TCS models shows that endogenous Treacle/ROS levels and strain background modulate severity and that reducing oxidative stress can ameliorate craniofacial defects; moreover, studies in craniofacial disorders using monozygotic twins support an epigenetic contribution to variable expressivity. Together, these observations highlight MZ twin pairs as a natural experiment to disentangle genotype-driven effects from non-genetic modifiers, with implications for counseling and future mechanistic studies like methylation and transcriptomic profiling. These data suggest that background-specific genetic modifiers regulate treacle protein levels and/or ROS homeostasis, thereby exerting profound effects on neural crest cell survival and craniofacial developmental outcomes [6,38,39].

In our cohort, Family 3 exemplifies this variable expressivity phenomenon, with severity scores ranging from mild (Patient 11: downward-slanting palpebral fissures and mild micrognathia only) to severe (Patient 9: microtia, external auditory canal atresia, profound bilateral hearing loss, downward-slanting palpebral fissures, malar and mandibular hypoplasia, dysphagia, and renal collecting system duplication). This striking intrafamilial heterogeneity reinforces the contribution of genetic and environmental modifiers beyond the primary inherited mutation. While the literature documents both autosomal dominant and autosomal recessive inheritance, rarer biallelic *POLR1D* presentations have also been described. Our extended pedigree, comprising five affected individuals across three generations, demonstrates unequivocal autosomal dominant inheritance with high penetrance and variable expressivity, clarifying this pattern in the Mexican population. This finding has immediate implications for genetic counseling: the recurrence risk is 50% for each offspring of an affected heterozygous individual, and careful clinical and molecular evaluation of ostensibly unaffected relatives is warranted, given the potential for subtle or minimal phenotypic expression in carriers [8,11,13].

Several patients in our cohort showed a heightened burden of perinatal complications, including neonatal respiratory distress and one neonatal death, largely driven by airway compromise secondary to micrognathia, which required emergent airway intervention and intensive postnatal care; these findings underscore the clinical value of timely prenatal detection and coordinated delivery planning to improve perinatal survival and neonatal outcomes. From the second trimester, prenatal ultrasound can identify hallmark TCS features and facilitate multidisciplinary planning involving neonatology, anesthesiology, and craniofacial surgery teams [40,41,42]; although fetal micrognathia has been associated with perinatal elevated perinatal mortality reported in selected series of severe micrognathia, outcomes improve when delivery occurs in tertiary centers with prepared airway teams [40,41,42]. In at-risk families, combining prenatal genetic testing (chorionic villus sampling or amniocentesis) with targeted WES and comprehensive sonographic assessment enables more precise risk stratification, supports informed perinatal decision-making, and allows teams to anticipate airway emergencies at delivery, thereby reducing morbidity and mortality while strengthening reproductive counseling and early-life management pathways [42,43].

The most frequent craniofacial and audiological findings in our cohort were auricular abnormalities (81%, 9/11 patients), downward-slanting palpebral fissures (72%, 8/11), malar hypoplasia (63%, 7/11), external auditory canal atresia (54%, 6/11), and conductive hearing loss of varying severity (54%, 6/11). This distribution generally aligns with reported frequencies in large international TCS series [44,45,46]. However, a notable finding was the complete absence of lower eyelid coloboma in our cohort, despite literature estimates of 54–69% prevalence in broader TCS populations. Several non-mutually exclusive mechanisms may explain this discrepancy: (1) ethnic and genetic background factors specific to the Mexican population; (2) the specific mutational spectrum in our families (predominantly *TCOF1* exon three and exon 24 variants, and *POLR1D* exon two mutations) which may differentially influence tissue-specific phenotypic expressivity; and/or (3) ascertainment or assessment factors, including the possibility of underreporting if comprehensive ophthalmologic examination was not uniformly documented across all patients. Notably, the high prevalence of auricular malformations (81%) and conductive hearing loss (54%) underscores the critical need for early audiological assessment and prompt hearing rehabilitation (including bone-anchored hearing devices or, when appropriate, cochlear implantation) to optimize speech and language development [44,45,46,47].

Variable expressivity, extensively documented in the TCS literature, is strikingly exemplified within Family 3 (Patients 7–11), where all five individuals carry the identical *POLR1D* frameshift deletion yet exhibit with widely divergent craniofacial manifestations and functional severity [48,49,50]. For example, Patient 9 presented with: microtia with external auditory canal atresia, profound bilateral sensorineural hearing loss, downward-slanting palpebral fissures, severe malar and mandibular hypoplasia with feeding dysfunction, dysphagia, and an incidental duplicated renal collecting system. In sharp contrast, Patient 11 exhibited only mild downward-slanting palpebral fissures and mild micrognathia, with no hearing loss or significant functional impairment. This striking intrafamilial phenotypic spectrum, spanning from subtle to severely disabling manifestations despite identical molecular etiology, highlights the critical interaction between the primary pathogenic variant and genetic/environmental modifier factors, and underscores the inherent challenge of accurate phenotypic prediction in genetic counseling even after molecular diagnosis confirmation [5,6,40,48,49,50].

Study limitations include both technical constraints of conventional WES and cohort ascertainment. In Patient 6, exome read-depth of ~0.46 relative to the diploid baseline indicated a heterozygous deletion ≥1 kb spanning *POLR1C* exons 1–2, illustrating WES’s reduced sensitivity for copy-number variation (CNV); notably, large deletions/duplications constitute ~5.2% of pathogenic *TCOF1* alleles and are readily missed by standard pipelines [40]. More comprehensive detection will require exome-based CNV callers with orthogonal confirmation (qPCR/MLPA) and, when indicated, WGS to resolve deep intronic, regulatory, and complex structural variants [8,50]. In parallel, our multigenerational, family-based design preferentially captures transmitted variants and under-represents de novo TCS, which limits generalizability, particularly for estimates of variant spectrum and expressivity. Future work should incorporate singleton/trio recruitment and population-representative cohorts, together with the above genomic enhancements, to obtain more unbiased estimates across the full clinical spectrum.

In silico computational predictors have emerged as powerful auxiliary tools for comprehensive variant characterization and pathogenicity assessment. These tools have been successfully applied to diverse aspects of genetic variant interpretation, including protein structure prediction with AlphaFold2 and splice-site disruption prediction with SpliceAI, Pangolin, and ESEFinder [51,52].

In silico analyses were used as supportive evidence for variant interpretation. Protein modeling (AlphaFold2) is consistent with loss-of-function for the two truncating alleles *TCOF1* p.(Arg77Profs?) (predicted ~173 aa vs. full-length treacle) and POLR1D p.(Gly99Alafs2) (~100 aa vs. 133 aa) by removing critical regions implicated in nucleolar localization and Pol I complex assembly. Splice predictors (SpliceAI, Pangolin) provided high-confidence disruption of the canonical +1 donor in the *TCOF1* splice variant (Family 1). Collectively, these concordant results fulfill PP3 (computational) as supporting evidence and complement the primary LOF framework (PVS1) used for classification. By refining localization of amino acid changes and assessing whether alterations disrupt functionally critical domains, this integrative approach frequently enables reclassification of initially ambiguous variants from VUS (variant of uncertain significance) status to likely pathogenic or pathogenic classification [53,54,55,56,57]. In silico analyses were interpreted solely as supporting evidence (PP3) and do not substitute for functional validation.

Comprehensive genetic counseling was provided to all patients and families, emphasizing the 50% recurrence risk for offspring of heterozygous affected individuals in autosomal dominant inheritance scenarios. This counseling was particularly valuable given that 6 of 11 patients (54.5%) were <18 years old, making information regarding reproductive planning, prenatal diagnostic options, and perinatal complication anticipation especially relevant for younger patients and their families. Molecular confirmation of pathogenic variants eliminates the diagnostic uncertainty inherent in clinical assessment alone. It provides families with a definitive molecular diagnosis, thereby ending the often-prolonged “diagnostic odyssey” that can span years and involve numerous inconclusive tests across multiple medical specialties. Although in family 3, the biological father of patients 7 and 8 was not available for molecular analysis, segregation among multiple affected relatives from the paternal branch supports autosomal dominant inheritance with a likely ancestral founder effect within this lineage.

Several patients in this cohort exhibited functional adaptations suggestive of upper airway and orofacial compromise. Patients 7 and 8 reported nocturnal breathing discomfort and adopted compensatory sleep postures, such as sleeping with two pillows. Although these behaviors are not diagnostic, they are clinically consistent with upper airway obstruction and align with the high prevalence of obstructive sleep apnea reported in individuals with Treacher Collins syndrome. Additionally, Patient 10 required a semi-solid diet due to a clinically narrow upper airway, reflecting functional limitations in feeding and mastication. These observations are concordant with previous reports describing impaired masticatory performance and altered oral function in Treacher Collins syndrome, in which craniofacial dysmorphology and airway restriction jointly impact sleep-related breathing and feeding efficiency. Collectively, these findings highlight the functional spectrum of the syndrome and underscore the importance of multidisciplinary evaluation beyond craniofacial morphology alone [58,59,60].

## 4. Materials and Methods

### 4.1. Patient Recruitment

This research was conducted in accordance with the Declaration of Helsinki and received approval from the Institutional Review Board of Facultad de Estudios Superiores Iztacala, UNAM (CE/FESI/072022/1537). Study participants were recruited through the Asociación Nacional Treacher Collins LIAM México, a patient advocacy organization, and from multiple tertiary-care institutions across Mexico providing specialized care to patients with rare craniofacial disorders. Written informed consent was obtained from all participants before enrollment in the study. Patients were included in the study if they met all of the following criteria: (1) age and sex unrestricted, willing to undergo comprehensive genetic testing and providing written informed consent; (2) Mexican birth or direct familial descent (Mexican-born parents or grandparents), ensuring population-specific genetic insights; and (3) clinical diagnosis was established by experienced clinicians based on established diagnostic criteria for TCS.

### 4.2. Molecular Analysis

#### 4.2.1. DNA Isolation

Genomic DNA was extracted from peripheral blood using the DNeasy Blood and Tissue Kit (QIAGEN, Hilden, Germany). The gDNA was quantified by fluorometry with the QuantiFluor dsDNA Kit and Quantus fluorometer (Promega, Madison, WI, USA). The integrity of dsDNA was analyzed by electrophoresis on a 0.8% agarose gel stained with SYBR Green dye. The purity of the dsDNA extraction was obtained with nanospectrometry (Implen, Munich, Germany) with the 260/280 nm ratio > 1.8 and <2.2.

#### 4.2.2. Whole-Exome Sequencing

gDNA samples were sent to Macrogen Inc. (Macrogen, Seoul, Republic of Korea) for WES. The dsDNA was fragmented and enriched for exon sequences using the Agilent SureSelect Human All Exon V6 Kit (Agilent Technologies, Santa Clara, CA, USA) [61] according to the manufacturer’s protocol. The kit has a target size of 60 Mb and covers 99% of exons according to databases such as RefSeq belonging to NCBI and OMIM_cds. WES was performed using paired-end 150 bp reads, targeting > 80% uniform coverage and a mean depth of 100×. The libraries were sequenced on an Illumina NovaSeq 6000 platform (Illumina, San Diego, CA, USA) [62].

### 4.3. Bioinformatics Analysis

Raw reads obtained from WES were subjected to quality analysis using FastQC v.0.11.9 (Babraham Institute, Cambridge, UK). Reads with a Phred score >30 were selected for bioinformatics analysis [63].

Once the sequences were selected, they were aligned to the reference genome GRCh38.P14 from NCBI (https://www.ncbi.nlm.nih.gov/datasets/genome/GCF_000001405.40/, accessed on 4 August 2025) using the Burrows–Wheeler Aligner (BWA) v. 0.7.13. Subsequently, they were sorted by coordinates and indexed with SAMtools v.1.13 software. Afterward, the data were recalibrated with the Genome Analysis Toolkit (GATK) programming framework v. 4.4.0.0 according best practices workflows suggested by the Broad Institute. The GATK Best Practices provide step-by-step recommendations for performing variant discovery analysis in high-throughput sequencing (HTS) data (https://gatk.broadinstitute.org/hc/en-us/articles/360035894711-About-the-GATK-Best-Practices, accessed on 4 August 2025). Following recalibration, variant calling was performed using GATK. Subsequently, single-nucleotide variants (SNVs) were identified, recalibrated, and compared against the dbSNP (https://www.ncbi.nlm.nih.gov/snp/, accessed on 4 August 2025), ClinVar (accessed on 4 August 2025), and 1000 Genomes databases (accessed on 4 August 2025). With the above, indels and SNVs were identified, recalibrated, and compared with the aforementioned databases. Exome-based SNV screening was performed using the Funcotator (FUNCtional annOTATOR) tool, belonging to GATK framework was used to annotate and identify variants. Variants were further annotated using in-house exome databases with 100 Mexican controls and the gnomAD (https://gnomad.broadinstitute.org/; accessed on 3 September 2025). Once the variants were filtered, they were searched in databases such as ClinVar, VarSome, OMIM, Franklin Genoox (https://franklin.genoox.com/clinical-db/home, accessed on 4 August 2025), InterVar, LOVD3, and PubMed (accessed on 18 January 2026) for correlations among genetic variants in genes, their modes of inheritance, phenotypes, and other characteristics [64,65,66,67,68,69,70,71,72,73,74,75,76,77].

Exon coordinate definition and coverage analysis for *POLR1C*. BAMs were aligned to GRCh38. Exon-level depth was extracted using SAMtools depth v1.22.1. Depth per exon was normalized to the per-exon cohort median (11 external WES + 3 internal controls), and a heterozygous-deletion threshold was defined as a normalized depth ratio of ≈0.5 relative to the diploid baseline. Read-depth values were derived from BAM alignments using the genomic coordinates of each exon; for every exon, the mean read depth was computed as the arithmetic mean of per-base coverage across the entire exon length, excluding positions with missing base calls (NA).

Exon-level coverage was performed against the MANE Select transcript for *POLR1C*, ENST00000642195.1 (Ensembl; Accessed 18 January 2026), on the GRCh38/hg38 reference genome. Genomic context: chromosome 6, positive strand; transcript span chr6:43,517,088–43,521,513. The transcript comprises nine exons, with exon boundaries (GRCh38) defined as:

Exon 1: 43,517,088–43,517,178
Exon 2: 43,517,305–43,517,377
Exon 3: 43,519,332–43,519,440
Exon 4: 43,519,705–43,519,838
Exon 5: 43,520,065–43,520,185
Exon 6: 43,520,274–43,520,427
Exon 7: 43,520,624–43,520,774
Exon 8: 43,520,931–43,521,048
Exon 9: 43,521,181–43,521,513

In parallel, exon coordinates were mapped to the corresponding cDNA intervals for interpretability and consistency with transcript-based analyses:

Exon 1: c.1–90; Exon 2: c.91–162; Exon 3: c.163–270; Exon 4: c.271–403; Exon 5: c.404–523; Exon 6: c.524–676; Exon 7: c.677–826; Exon 8: c.827–943; Exon 9: c.944–1275.

Regional coverage dropout and exon boundaries were inspected in IGV. Results were visualized as bar plots generated with ggplot2, enabling comparison of relative exon coverage across samples.

### 4.4. Variant Filtering and Selection

To identify pathogenic SNVs, each candidate variant was evaluated in databases such as ClinVar (https://www.ncbi.nlm.nih.gov/clinvar/) (accessed on 18 September 2025 and 18 January 2026), 1000 Genomes (https://www.internationalgenome.org/) (accessed on 18 September 2025 and 18 January 2026), VarSome (https://varsome.com/) (accessed on 18 September 2025 and 18 January 2026), Franklin Genoox (https://franklin.genoox.com/clinical-db/home) (accessed on 18 September 2025 and 18 January 2026), InterVar: Guideline (https://wintervar.wglab.org/) (accessed on 18 September 2025 and 18 January 2026), LOVD v.3.0 (https://www.lovd.nl/3.0/home) (accessed on 18 September 2025 and 18 January 2026), and dbSNP (https://www.ncbi.nlm.nih.gov/snp/) (accessed on 18 September 2025 and 18 January 2026).

Computational tools and sequence conservation analysis used from various in silico predictors were MutationTaster2 v. 2025 (https://www.mutationtaster.org/, accessed on 4 August 2025), ConSurf Server (https://consurfdb.tau.ac.il, accessed on 4 August 2025), REVEL, SIFT, and CADD. All variants with an allele frequency > 0.01% of the worldwide allele frequency or population frequency of American admixture, that do not present the specific mode of inheritance for the disorder with the gene in question, or that present <2 manifestations related to the affected gene, were excluded [45,46,47].

Once the probable variant causing the patient’s clinical manifestations was identified, visual verification was performed with the Integrative Genome Viewer (IGV). Allele frequencies were obtained from gnomAD v4.1.0 (accessed on 3 September 2025 and 18 January 2026). To assess the functional impact of genetic variants, scores from the in silico predictors REVEL, SIFT, CADD, SpliceAI, Pangolin, and PrimateAI were used [78,79,80,81,82,83,84].

All results were classified according to the guidelines for the interpretation of genetic variants proposed by the American College of Medical Genetics and Genomics (ACMG) and the Association for Molecular Pathology (AMP), as well as the Best Practice Guidelines for Variant Classification in Rare Disease [85,86].

### 4.5. PCR and Sanger Sequencing

Genetic variant validation and familial segregation studies were performed by Sanger sequencing. Specific oligonucleotides were designed to flank the region containing the patients’ genetic variants. For *TCOF1*, specific primers for exon 3 and exon 24 were designed. For the *POLR1D* gene, primers were designed to flank exon 2 using the Primer3Plus website. These reactions were amplified from the genomic DNA of patients and family members, respectively, by conventional PCR. The primer sequences were:

Gene: *TCOF1*

- Exon 24: Fw 5′-GCCTCTGTTTCCCCAGAAAA-3′ and
  Rev 5′-ACATGGGAGGAATGAGACCA-3′
- Exon 3: Fw 5′-CACATTGCCTTTAAGAGCTG-3′ and
  Rev 5′-ACGGAGGAAGGGCTCAAATA-3′

Gene: *POLR1D*

- Exon 2: Fw 5′-AGGAAGACAGCCCTGGAAAT-3′ and
  Rev 5′-GAGGTTCTTGCAGAGATTCC-3′

PCR amplicons were purified using the AMPure XP fragment purification kit (Beckman Coulter, Brea, CA, USA). Finally, they were used for Sanger sequencing to validate the genetic variant [87].

### 4.6. Three-Dimensional Protein Modeling

Three-dimensional protein structures of wild-type and mutant treacle and POLR1D were computationally predicted using AlphaFold2 (AF2) via the ColabFold interface v. 1.5.5, predictions were generated with ColabFold/AF2; structural outputs (pLDDT, model confidence) were used descriptively to contextualize variant impact. Mutant sequences were generated by computationally introducing patient-specific pathogenic variants into wild-type cDNA sequences using the Biomodel (https://biomodel.uah.es/, accessed on 4 August 2025) platform, which automated transcription and translation to amino acid sequences. AF2 predictions were processed with 5 recycling cycles and assigned confidence metrics (pLDDT scores); regions scoring ≥70 were classified as high-confidence structural predictions. Predicted structures were validated using Ramachandran plots to assess backbone dihedral angle distributions, with acceptance criteria requiring >97% of amino acids in allowed conformational regions, consistent with well-refined crystallographic structures. High-quality structures were visualized using UCSF Chimera v. 1.11.1 and compared between wild-type and mutant variants, evaluating effects on secondary structure stability, domain architecture, protein–protein interaction interfaces critical for nucleolar localization and ribosome biogenesis [29,74,88,89,90,91].

### 4.7. In Silico Predictors

For in silico predictors, the scores obtained when entering the repository pages of SpliceAI (https://spliceailookup.broadinstitute.org/), Pangolin (https://github.com/tkzeng/Pangolin, accessed on 4 August 2025), CADD, REVEL, SIFT, MutationTaster2, and ESEFinder 3.0 (https://esefinder.ahc.umn.edu/cgi-bin/tools/ESE3/esefinder.cgi, accessed on 4 August 2025) were used [51].

## 5. Conclusions

This study identified three previously unreported pathogenic variants in three unrelated Mexican families achieving molecular genetic diagnoses in 10 of 11 patients (90.9% diagnostic yield) through comprehensive whole-exome sequencing. All three identified variants meet stringent ACMG/AMP criteria for pathogenic classification, supported by rigorous application of ACMG guidelines, comprehensive segregation analysis across affected relatives, convergent predictions from independent in silico tools, and structural modeling demonstrating loss-of-function consequences. Collectively, these variants constitute the first molecularly confirmed and characterized TCS cases reported from the Mexican population, thereby filling a significant gap in geographic and ethnic representation within international genetic variant repositories and clinical TCS literature.

Our comprehensive clinical–molecular analysis demonstrates that genotype alone does not fully predict phenotype in TCS, even when examining families with identical pathogenic variants. The striking variable expressivity observed within Family 3 (five affected individuals with identical *POLR1D* deletion yet ranging from mild to severe manifestations) exemplifies the complex interaction between primary genetic variants, genetic modifiers, and developmental stochasticity in determining disease severity and craniofacial outcomes. This observation underscores the importance of integrating molecular findings with detailed clinical characterization to provide meaningful genetic counseling and prognostic information to affected families.

## Figures and Tables

**Table 1:** Clinical Characteristics of Patients with TCS. Background and detailed clinical presentation of each patient are described. TPL: Threatened Preterm Labor, TA: Threatened Abortion, DTETB: Delayed Tooth Eruption of the Temporal Bone, BB: Brachycephaly at Birth. ●: Present; ―: Absent.

Clinical Features	P1	P2	P3	P4	P5	P6	P7	P8	P9	P10	P11
**Affected gene**	*TCOF1*	*TCOF1*	*TCOF1*	*TCOF1*	*TCOF1*	*POLR1C*	*POLR1D*	*POLR1D*	*POLR1D*	*POLR1D*	*POLR1D*
**Gender**	Male	Male	Female	Male	Male	Male	Female	Female	Female	Female	Female
**Age**	8	11	38	15	55	3	7	7	34	25	60
**Perinatal Complications**	TLP	Placental Abruption	Unknown	3 TA	Unknown	―	―	―	―	Unknown	―
**Intubation or Tracheostomy in neonates**	―	●	―	―	Unknown	―	―	―	―	―	―
**Nasogastric Tube or Gastrostomy in neonates**	●	―	―	―	Unknown	―	―	―	―	―	―
**Total Surgeries**	0	0	18	0	>20	0	1	2	11	0	0
**Microcephaly**	―	―	―	―	―	―	BB	BB	―	―	―
**Cleft Palate**	―	―	―	―	―	―	Submucosal	Submucosal	―	―	―
**Preauricular Hair Displacement**	―	―	―	―	●	●	―	―	―	―	―
**Auricular Pavilion Abnormalities (Shape, Size, Rotation)**	●	●	●	―	●	●	●	●	●	●	―
**Atresia of the External Auditory Canal**	●	●	―	―	●	●	―	―	●	●	―
**Microtia**	●	●	●	―	―	●	―	―	●	―	―
**Hearing Loss**	Severe hearing loss in the right ear and moderate hearing loss in the left ear	Severe hearing loss in the right ear and moderate hearing loss in the left ear	Unknown	Normal	Unknown	Severe hearing loss in the right and left ear	Severe hearing loss in the right and left ear	Severe hearing loss in the right and left ear	Severe hearing loss in the right and left ear	Normal	Normal
**Hearing Aid**	●	●	●	―	―	―	●	―	●	―	―
**Coloboma (notching) of Lower Lid**	―	―	―	―	―	―	―	―	―	―	―
**Downslanted Palpebral Fissures**	●	●	●	●	●	―	●	―	●	●	●
**Malar Hypoplasia/Hypoplasia of Zygomatic Bones**	●	●	●	●	●	―	●	―	●	―	―
**Mandibular Hypoplasia/Micrognathia**	●	Retromicrognathia	●	●	●	●	●	●	●	●	●
**Dental Abnormalities**	DTETB	Multiple caries-like lesions	―	―	―	●	●	●	―	―	―
**Chewing/Swallowing Problems**	―	―	―	―	●	―	―	―	●	―	―
**Delayed Speech Development**	●	●	―	●	―	●	●	●	●	―	―
**Intellectual Disability**	―	● Both	―	―	―	●	―	―	―	―	―
*Other Features*	―	*Hypoxic-Ischemic Encephalopathy Grade I at Birth*	*Postpartum Hemorrhage in her Second Pregnancy*	*Factor XII Coagulation Deficiency and Vitamin D Deficiency*	―	―	*Bilateral Malleus and Incus Malformation. Hypoplasia of the Left Tympanic Cavity*	*Left Mastoid Hypoplasia, Adenoid Hypertrophy Grade III*	*Double Renal Collecting System*	―	―

## Data Availability

Variant-level data (HGVS, GRCh38 coordinates, ACMG/AMP evidence) and Supplementary Tables are provided with the article. Raw WES data (FASTQ/BAM/VCF) contain potentially identifiable information and cannot be deposited publicly due to consent restrictions. The data presented in this study are available on request from the corresponding author due to Controlled access (institutional/ethics agreement).

## Supplementary Materials

The following supporting information can be downloaded at: https://www.mdpi.com/article/10.3390/ijms27041891/s1.

## Abbreviations

The following abbreviations are used in this manuscript:

TCS	Treacher Collins syndrome
WES	Whole-exome sequencing
NGS	Next-generation sequencing
FD	Facial dysostoses
MFD	Mandibulofacial dysostoses
AFD	Acrofacial dysostoses
AD	Autosomal dominant
AR	Autosomal recessive
NCC	Neural crest cells
SNV	Single-nucleotide variants
GATK	Genome Analysis Toolkit
ACMG	American College of Medical Genetics and Genomics
AMP	Association for Molecular Pathology
RMSD	root-mean-square deviation
PTC	Premature termination codons
